# Theoretical Modeling and Experimental Verification of the Bending Deformation of Fiber Metal Laminates

**DOI:** 10.3390/ma16093486

**Published:** 2023-04-30

**Authors:** Yizhe Chen, Zhuoqun Wang, Yi Lin, Hui Wang, Lin Hua

**Affiliations:** 1 Hubei Key Laboratory of Advanced Technology for Automotive Components, Wuhan University of Technology, Wuhan 430070, China; 2 Hubei Collaborative Innovation Center for Automotive Components Technology, Wuhan 430070, China; 3 Jiangsu Xinyang New Material Co., Ltd., Yangzhou 225000, China; 4 Hubei Engineering Research Center for Green & Precision Material Forming Technology, Wuhan 430070, China; 5 Hubei Longzhong Laboratory, Xiangyang 441000, China

**Keywords:** fiber metal laminates, elastic-plastic bending, computational modelling, mechanical properties

## Abstract

Fiber metal laminates have been widely used as the primary materials in aircraft panels, and have excellent specific strength. Bending deformation is the most common loading mode of such components. An accurate theoretical predictive model for the bending process for the carbon reinforced aluminum laminates is of great significance for predicting the actual stress response. In this paper, based on the metal-plastic bending theory and the modified classical fiber laminate theory, a modified bending theory model of carbon-fiber-reinforced aluminum laminates was established. The plastic deformation of the thin metal layer in laminates and the interaction between fiber and metal interfaces were considered in this model. The bending strength was predicted analytically. The FMLs were made from 5052 aluminum sheets, with carbon fibers as the reinforcement, and were bonded and cured by locally manufacturers. The accuracy of the theory was verified by three-point bending experiments, and the prediction error was 8.4%. The results show that the fiber metal laminates consisting of three layers of aluminum and two layers of fiber had the best bending properties. The theoretical model could accurately predict the bending deformation behaviors of fiber metal laminates, and has significant value for the theoretical analysis and performance testing of laminates.

## 1. Introduction

At present, aluminum alloy and carbon fiber composites are widely used in many industries. Fiber metal laminate (FMLs) is a hybrid laminate made of alternating metal and fiber layers, which is then solidified under certain temperatures and pressures [1,2]. FMLs integrate the characteristics of traditional fiber composite materials and metal materials. FMLs have the advantages of high specific strength and stiffness, an excellent fatigue response, corrosion resistance, and good design flexibility. Due to the addition of the metal layer, the defect of weakness under the in-plane compression of fiber-reinforced polymers (FRPs) is effectively improved, and FMLs show strong in-compression mechanical performance [3,4]. In some specific cases, FMLs also have the advantages of low electrical conductivity and thermal conductivity. Therefore, FMLs have become a popular engineering material that is widely used in aerospace, automotive, marine, construction, and other industries [5,6]. The characteristics of mechanical properties under external loads have recently become a research hotspot [7,8].

FMLs are the primary materials of critical components such as the panels and tail fins of some important aircraft, such as the Airbus A380. Bending is a common form of loading throughout the whole service process. FMLs exhibit enhanced in-compression mechanical performance, which is attributed to the addition of metal ply. The research group led by Professor Tao Jie of Nanjing University of Aeronautics and Astronautics is at the top international level in this field and has conducted a significant amount of work. Interface behavior has a significant influence on bending behavior. Li et al. [9] studied the influence of the interface performance on the mechanical properties of glass-fiber-reinforced aluminum laminates (Glare). They found that the interface properties have a more significant influence on bending strength than tensile strength. Bellini et al. [10] studied the influence of adhesives and single or double metal plates on the bending properties of FMLs. They found that the bending strength of the materials was improved in the absence of adhesive layers and single metal plates. Ostapiuk et al. [11] studied the cracking and failure mechanisms of FMLs. They found that the existence of an aluminum layer effectively prevented the failure of FMLs during the bending process. Therefore, the interface property is an essential factor affecting the bending properties of FMLs.

Many factors affect the interface performance of laminates, and the lamination of FMLs is particularly significant due to the lamination structure. Lamination determines the number of interfaces and thus influences the bending behavior. It includes the layering angle and structure and the lamination thickness. Carrillo et al. [12] studied the influence of the layering angle on the flexural performance of polypropylene self-reinforced aluminum alloy laminates. They found that the layering angle had little influence on the FMLs’ performance. The layer structure influences the bending properties. Fu et al. [13] studied the influence of the layering structure on the bending performance of carbon-fiber-reinforced aluminum laminates (CARALL). They found that 2/1 FMLs easily transferred loads under bending conditions without interfacial delamination. The 5/4 FMLs exhibit evident delamination in the outermost layer under bending deformation. Ritesh et al. [14] studied the effect of material geometry in terms of material thickness on the mechanical properties of glass-fiber-reinforced isophthalic polyesters. They found that the material’s thickness is a significant factor affecting its mechanical properties. The above studies analyzed the effect of lamination on the bending properties of laminates from various perspectives. However, insufficient research exists regarding the influence of lay-ups on the bending performance based on automobile and transportation equipment.

The research on the bending properties of FMLs is mainly based on experiments, but these experiments are usually cumbersome and costly. Therefore, it is of great significance to carry out theoretical research into the properties of FMLs to improve efficiency and reduce costs. At present, the theories of FMLs are mainly based on the classical laminates theory, or else use finite element simulation analysis to predict the bending properties of laminates. Rajabi et al. [15] carried out finite element simulation of FMLs using Timoshenko beam theory and predicted the three-point bending properties of the materials. Li et al. [16] found that changing the span thickness ratio of FMLs in failure mode significantly impacted their bending properties. Ud Din, I. et al. [17] developed an elastoplastic continuum damage model, which uses Puck’s theory in the case of plane stress as the meso-damage initiator for the corresponding mode of failure and the one-parameter plasticity potential with isotropic strain hardening. The prediction of mechanical behaviors and subsequent failure has been significantly improved from the previous investigations by employing coupled damage evolution and plasticity. Subsequent studies [18] have also shown that, in the presence of compression, more accurate results can be obtained by using the Puck theory. Lee et al. [19] established a bending finite element model of FMLs to analyze the bending performance of the laminates according to thickness. Dariushi et al. [20] used geometric nonlinear classical theory to predict the force-deflection behavior in the bending deformation of FMLs. They obtained the displayed calculation formula between deflection and force. Jin et al. [21] established a three-dimensional finite element constitutive model to analyze the mechanical behavior of FMLs under bending deformation. It predicted the damage of the metal layers, fiber layers, and interface delamination. However, due to the complexity of the process of preparing FMLs, the existing bending theory prediction was mainly based on the classical lamination theory of fibers and the finite element simulation. The plastic strain of the metal layer and the interaction between the metal and fiber interface were not considered at the same time, which made the prediction method more complicated and less accurate. Therefore, there is an urgent need to establish a more accurate model to predict the variation in bending stress of fiber-metal laminates and to analyze the influence of process parameters on the materials’ properties.

In this paper, a modified bending theory model of carbon-fiber-reinforced aluminum laminates (CARALL) was established. Based on the metal-plastic bending theory and the modified classical fiber laminate theory, the bending process carried out in experiments was analyzed. The bending strength of CARALL was predicted using the proposed method. Bending experiments on CARALL with different layers were carried out. The accuracy of the theoretical model was verified by combining the experimental results and theoretical prediction results. Finally, based on the theoretical model, the influence of process parameters on the bending properties of laminates was analyzed.

## 2. Materials and Methods

In this paper, the aluminum alloy is 5052, which is a type of Al-Mg alloy. The density is 2.68 g/cm^3^, and the thickness is 0.5 mm. The carbon fiber is an orthogonal carbon fiber/epoxy resin prepreg produced by Weihai Guangwei Composite Material Co., Ltd., Weihai, China. The resin content is 40% and the thickness is 0.2 mm. The grade of the epoxy resin is R5600W3K. The grade of the carbon fiber is SYT35/3K. The basic parameters of the material are provided by the manufacturer. The performance parameters of carbon fiber are shown in Table 1. The primary performance parameters of carbon fiber and epoxy resin are shown in Table 2.

**Figure 1:**
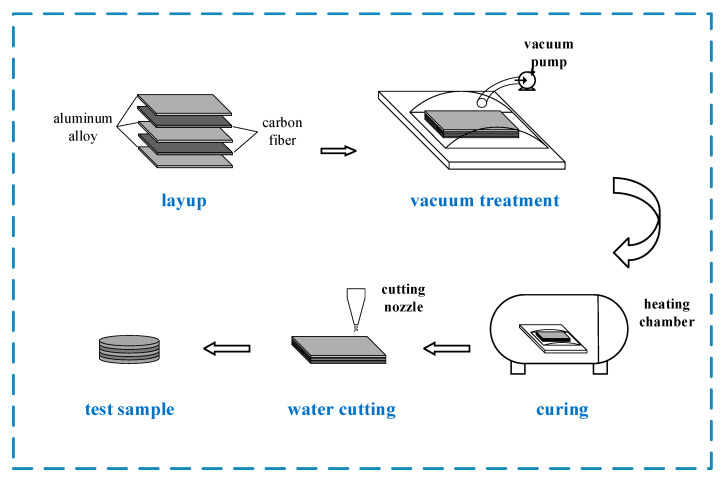
CARALL preparation process flow chart.

The preparation flow chart for the CARALLs is shown in Figure 1.The CARALLs were prepared using the following steps. First, the surface roughness of the aluminum alloy layer was improved by phosphoric acid anodic oxidation before laminating. This increases the bonding strength between the aluminum alloy and the carbon fiber, which improves the properties of the laminates. Then, a 100 × 100 mm aluminum alloy sheet and a 100 × 100 mm carbon fiber prepreg were laid alternatingly, with the aluminum alloy as the outer layer and the carbon fiber as the inner layer. The 3 lay-ups are 2/1, 3/2, and 4/3. The direction of the carbon fiber prepreg was consistent with the rolling direction of the aluminum alloy. When the paving was completed, a barrier film was placed on the surface of the laminate and a breathable felt was attached; then, the entire laminate was placed in a vacuum bag and vacuumed to extract any air that remained inside. After the completion of the lamination, the pre-cured laminates were put into the heating chamber for heating and curing. They were heated to 80 °C for 30 min and then 130 °C for 60 min. Finally, the laminates were taken out and cooled to room temperature to obtain the cured CARALLs.

**Figure 2:**
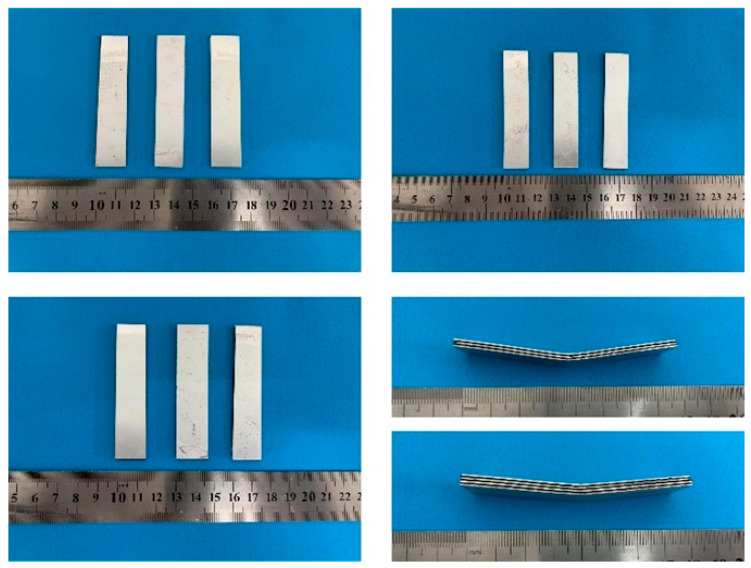
Bending specimens and the side views of different lay-ups.

The bending test standards refer to ASTM D790-2017. The bending sample size is 70 × 15 mm. Laser cutting produces a thermal effect on the laminate. Therefore, water cutting was used to prepare the sample of CARALLs. The cutting sample is shown in Figure 2.

**Figure 3:**
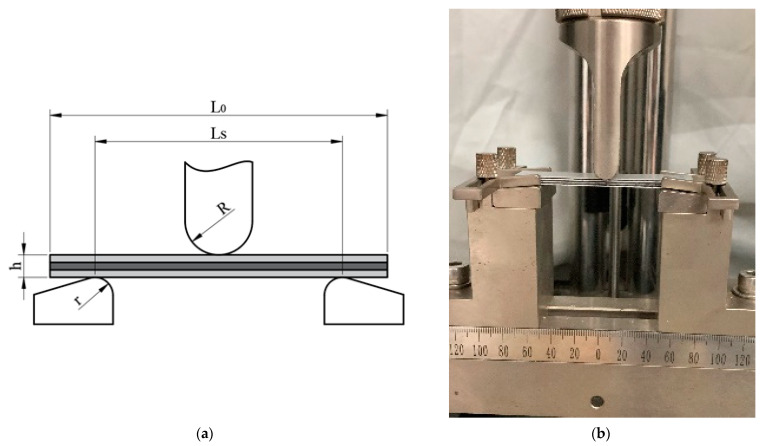
Schematic diagram of the bending test dimensions and instrument; (**a**) three-point bending and (**b**) bending test.

The relevant dimensions and test instruments for the bending test are shown in Figure 3. The instrument used in this bending test is the SANS microcomputer-controlled electronic universal testing machine, grade CMT5205, produced by MTS SYSTEMS (Shanghai, China) Co., Ltd. In Figure 3, $L_{0}$ is the sample length, $L_{s}$ is the span, h is the sample thickness, the supporting fillet radius r is 2 mm, the punch radius R is 5 mm, and the punch displacement speed is 2 mm/min. There were three groups of bending samples of CARALLs with different lay-ups, and the number of specimens for each material is three. The final test results were averaged to ensure consistency.

## 3. Theoretical Modeling

**Figure 4:**
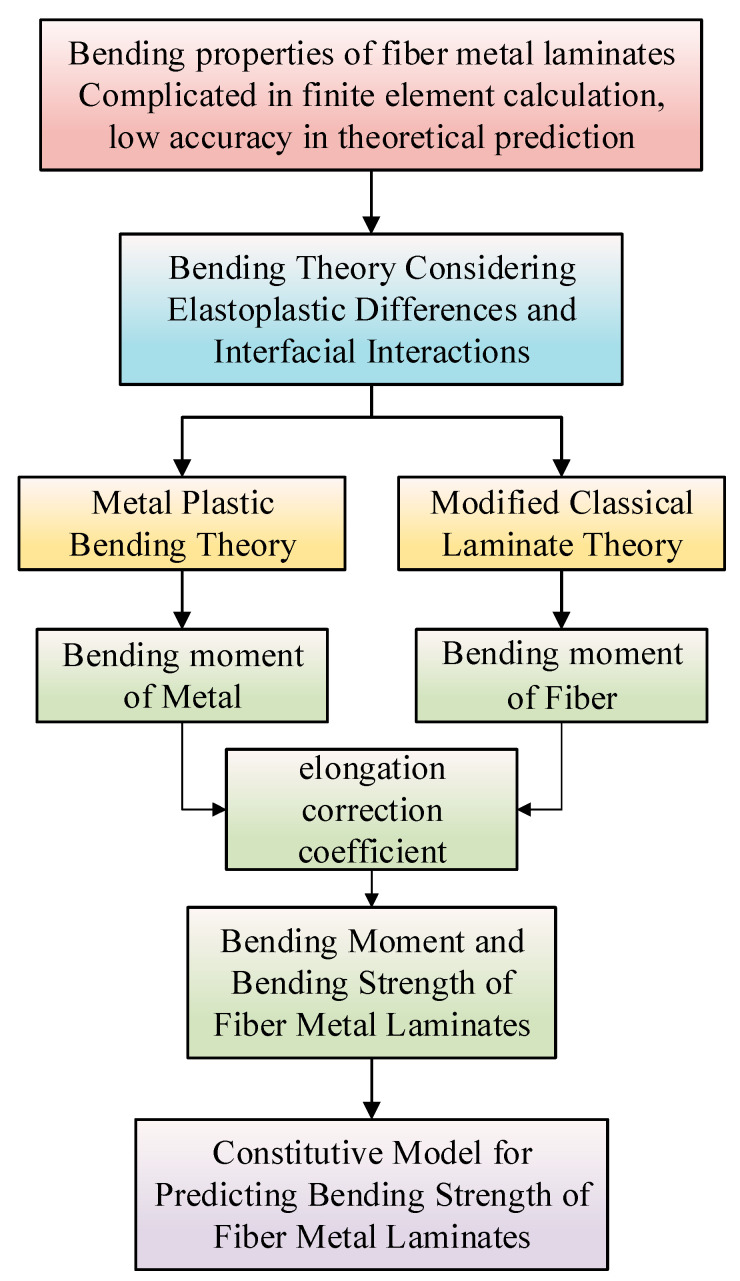
Flow chart of the theoretical modeling of the bending strength of FMLs.

A thin CARALL is usually used as a cover. In this paper, the bending deformation behavior of CARALLs was studied. The laminate was made of an aluminum alloy and a carbon fiber. The aluminum alloy has good plasticity, while carbon fiber has almost no plasticity. Therefore, a theoretical analysis of the aluminum alloy and the carbon fiber characteristics was carried out. Then, the bending theory of FMLs was constructed. Finally, a model for calculating the bending stress of FMLs was obtained, considering the elastic–plastic difference and the interface effect. The modeling process is shown in Figure 4.

### 3.1. Metal Layer Theory

**Figure 5:**
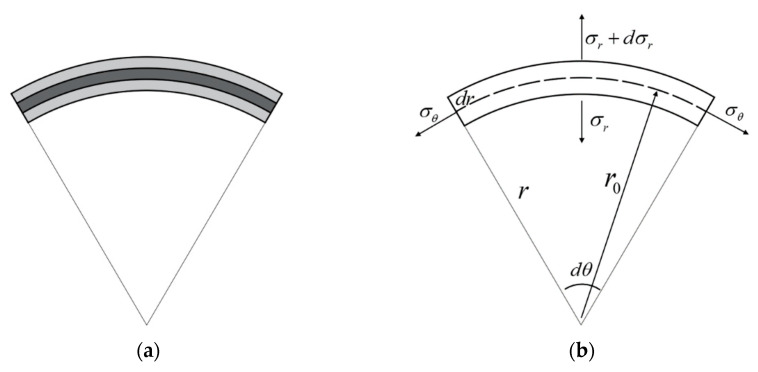
(**a**) Diagram of laminate bending; (**b**) diagram of bending deformation stress.

The stress and strain in the width direction could be ignored when the laminates were subjected to a three-point bending state [22]. The three-point bending analysis of the laminates could be regarded as the plane stress state. The micro-element deformation state under the action of pure bending is shown in Figure 5.

Where $\sigma_{r}$ is the radial stress, $\sigma_{\theta }$ is the circumferential stress, the angle of the cell is dθ, and the radius is r. According to the stress balance Equation (1) and the mises yield condition criterion (2), the relationship between radial stress and circumferential stress can be obtained as follows:

(1) $$r\frac{d\sigma_{r}}{dr}=\sigma_{\theta }-\sigma_{r}$$

(2) $$\sigma_{\theta }-\sigma_{r}=\frac{2}{\sqrt{3}}\bar{\sigma }$$

The equivalent stress $\bar{\sigma }$ of the material is a function of the equivalent strain; meanwhile, in pure bending, the equivalent strain $\bar{\varepsilon }$ depends on the radius *r*. Therefore, the equal effect is a function of the radius.

(3) $$\bar{\sigma }=\bar{\sigma }\left(\bar{\varepsilon }\right)=\bar{\sigma }\left(r\right)$$

The radial stress can be obtained by integrating the stress balance Equation (1):

(4) $$\sigma_{r}=\int_{r_{i}}^{r}\frac{2}{\sqrt{3}}\cdot \frac{\bar{\sigma }\left(r\right)}{r}dr$$

For metals, elasto-plastic with a strain hardening constitutive model is used. Its stress–strain relationship is shown in Formula (5). *K* is the hardening coefficient. *n* is the hardening index. $d\varepsilon_{r}$ is the radial strain increment. $d\varepsilon_{\theta }$ is the circumferential strain increment. $d\varepsilon_{s}$ is the axial strain increment. According to the incompressible condition of plane strain and plastic deformation, the sum of radial and axial strain is also zero. Therefore, the equivalent strain increment can be obtained by combining the plastic increment theory.

(5) $$\bar{\sigma }=K\cdot {\bar{\varepsilon }}^{n}$$

(6) $$d\bar{\varepsilon }=\pm \sqrt{\frac{2}{3}\left(d\varepsilon_{r}^{2}+d\varepsilon_{\theta }^{2}+d\varepsilon_{s}^{2}\right)}$$

(7) $$\bar{\varepsilon }=\frac{2}{\sqrt{3}}\int \left|d\varepsilon_{\theta }\right|$$

The following formula can be obtained by combining the above formula with the stress balance Equation (1), the mises yield condition (2) and the equivalent stress (5) and strain (7):

(8) $$r\frac{d\sigma_{r}}{dr}=\frac{2}{\sqrt{3}}k\cdot {\left(\frac{2}{\sqrt{3}}\int \left|d\varepsilon_{\theta }\right|\right)}^{n}$$

During the bending process, assuming that the length and thickness of the laminates before bending are $l_{0}$ and $t_{0}$, respectively, the bending radian of the plate is α, and the layer of constant length is located at the position of “$r=r_{0}$”. The circumferential strain can be expressed as:

(9) $$\varepsilon_{\theta }=\ln\left(l/l_{0}\right)=\ln\left(r\alpha /l_{0}\right)=\ln\left(r\alpha /r_{0}\alpha \right)=\ln\left(r/r_{0}\right)$$

Therefore, the equivalent stress and radial stress can be expressed as:

(10) $$\bar{\sigma }=k\cdot {\bar{\varepsilon }}^{n}=k\cdot {\left[\frac{2}{\sqrt{3}}\ln\left(\frac{r}{r_{0}}\right)\right]}^{n}$$

(11) $$r\frac{d\sigma_{r}}{dr}=\frac{2}{\sqrt{3}}k\cdot {\left[\frac{2}{\sqrt{3}}\ln\left(\frac{r}{r_{0}}\right)\right]}^{n}=\frac{2^{n+1}}{\sqrt{3^{n+1}}}k\cdot \ln^{n}\left(\frac{r}{r_{0}}\right)$$

(12) $$\sigma_{r}=\frac{2^{n+1}}{\sqrt{3^{n+1}}}k\int_{r_{i}}^{r}\frac{\ln^{n}\left(r/r_{0}\right)}{r}dr=\frac{2^{n+1}}{\sqrt{3^{n+1}}}k\int_{r_{i}}^{r}\ln^{n}\left(r/r_{0}\right)d\left[\ln\left(r/r_{0}\right)\right]$$

During the bending process, the direction of stress varies with the bending radius. The outer layer is subjected to tensile stress and the inner layer is subjected to compression stress. Therefore, piecewise analysis of stress is needed, according to the radius. Assuming that the radius of the neutral layer is $r_{n}$, the radius of the outermost and innermost layers of the laminate are $r_{y}$ and $r_{i}$, respectively. The radial stress is segmented as follows:

(13) $$\sigma_{r}=\{\begin{array}{c} \frac{2^{n+1}}{\sqrt{3^{n+1}}}\cdot \frac{k}{n+1}\left[\ln^{n+1}\left(r/r_{0}\right)-\ln^{n+1}\left(r_{y}/r_{0}\right)\right],(r_{0}\leq r\leq r_{y})\\ \frac{2^{n+1}}{\sqrt{3^{n+1}}}\cdot \frac{k}{n+1}\left\{-{\left[-\ln\left(r/r_{0}\right)\right]}^{n+1}-{\left[\ln\left(r_{y}/r_{0}\right)\right]}^{n+1}\right\},(r_{n}\leq r\leq r_{0})\\ \frac{2^{n+1}}{\sqrt{3^{n+1}}}\cdot \frac{k}{n+1}\left\{{\left[-\ln\left(r/r_{0}\right)\right]}^{n+1}-{\left[-\ln\left(r_{i}/r_{0}\right)\right]}^{n+1}\right\},(r_{i}\leq r\leq r_{n}) \end{array}$$

The circumferential stress $\sigma_{\theta }$ can be further calculated from the mises yield condition:

(14) $$\sigma_{\theta }=\{\begin{array}{c} \frac{2^{n+1}}{\sqrt{3^{n+1}}}k\cdot \ln^{n}\left(r/r_{0}\right)\left[1-\frac{\ln\left(r/r_{0}\right)}{n+1}\right]+\frac{2^{n+1}}{\sqrt{3^{n+1}}}\cdot \frac{k}{n+1}\ln^{n+1}\left(r_{y}/r_{0}\right),(r_{0}\leq r\leq r_{y})\\ \frac{2^{n+1}}{\sqrt{3^{n+1}}}k\cdot {\left[-\ln\left(r/r_{0}\right)\right]}^{n}\left[1+\frac{-\ln\left(r/r_{0}\right)}{n+1}\right]+\frac{2^{n+1}}{\sqrt{3^{n+1}}}\cdot \frac{k}{n+1}\ln^{n+1}\left(r_{y}/r_{0}\right),(r_{n}\leq r\leq r_{0})\\ -\frac{2^{n+1}}{\sqrt{3^{n+1}}}k\cdot {\left[-\ln\left(r/r_{0}\right)\right]}^{n}\left[1-\frac{-\ln\left(r/r_{0}\right)}{n+1}\right]-\frac{2^{n+1}}{\sqrt{3^{n+1}}}\cdot \frac{k}{n+1}{\left[-\ln\left(r_{i}/r_{0}\right)\right]}^{n+1},(r_{i}\leq r\leq r_{n}) \end{array}$$

The bending moment of the metal layer is shown below:

(15) $$\begin{array}{l} M=\int_{r_{i}}^{r_{y}}\sigma_{\theta }rdr=\int_{r_{0}}^{r_{y}}\left\{\frac{2^{n+1}}{\sqrt{3^{n+1}}}kr\cdot \ln^{n}\left(r/r_{0}\right)\left[1-\frac{\ln\left(r/r_{0}\right)}{n+1}\right]+\frac{2^{n+1}}{\sqrt{3^{n+1}}}\cdot \frac{kr}{n+1}\ln^{n+1}\left(r_{y}/r_{0}\right)\right\}dr+\\ \int_{r_{n}}^{r_{0}}\left\{\frac{2^{n+1}}{\sqrt{3^{n+1}}}kr\cdot {\left[-\ln\left(r/r_{0}\right)\right]}^{n}\left[1+\frac{-\ln\left(r/r_{0}\right)}{n+1}\right]+\frac{2^{n+1}}{\sqrt{3^{n+1}}}\cdot \frac{kr}{n+1}\ln^{n+1}\left(r_{y}/r_{0}\right)\right\}dr+\\ \int_{ri}^{r_{n}}\left\{-\frac{2^{n+1}}{\sqrt{3^{n+1}}}kr\cdot {\left[-\ln\left(r/r_{0}\right)\right]}^{n}\left[1-\frac{-\ln\left(r/r_{0}\right)}{n+1}\right]-\frac{2^{n+1}}{\sqrt{3^{n+1}}}\cdot \frac{kr}{n+1}{\left[-\ln\left(r_{i}/r_{0}\right)\right]}^{n+1}\right\}dr \end{array}$$

### 3.2. Modified Fiber Layer Theory

The classical lamination theory is the most commonly used and effective method for fiber-reinforced composite laminates. The classical laminate theory is only applicable to unidirectional fiber composites and multidirectional laminates. Further improvement is needed when it is applied to complex braided fiber composites. Therefore, this study used the classical lamination theory and modified the carbon fiber layer theory to predict the stress and strain relationship of the fiber layer.

**Figure 6:**
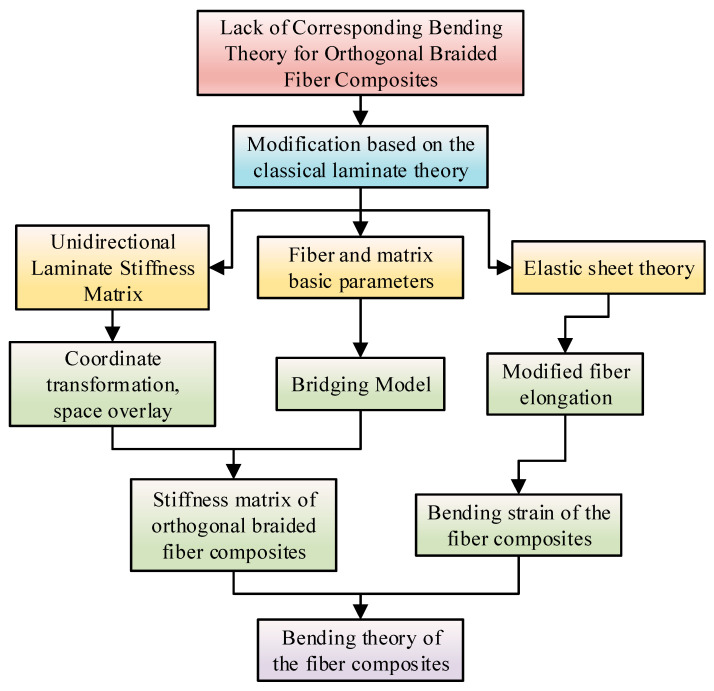
Flow chart of the theoretical modeling of the fiber layer.

The application of the orthogonal braided carbon fiber laminate was increasing gradual. It was necessary to carry out a theoretical analysis of its mechanical properties. Therefore, the analytical model of the orthogonal braided laminate was established. The derivation process of the bending theory of the orthogonal braided carbon fiber laminate is shown in Figure 6.

First, according to the classical laminate theory, the flexibility matrix $\left[S_{ij}\right]$ of the unidirectional fiberboard is shown as follows:

(16) $$\left[S_{ij}\right]=\left[\begin{array}{ccc} \frac{1}{E_{11}} & -\frac{\nu_{12}}{E_{11}} & 0\\ -\frac{\nu_{12}}{E_{11}} & \frac{1}{E_{22}} & 0\\ 0 & 0 & \frac{1}{G_{12}} \end{array}\right]$$

where $E_{11}$, $E_{22}$, $G_{12}$, and $\nu_{12}$ are the axial modulus, transverse modulus, shear modulus, and Poisson’s ratio of fiber laminates, respectively.

The carbon-fiber-reinforced epoxy resin composite plate with an orthogonal weaving structure can be approximately regarded as two equal-sized unidirectional carbon-fiber-reinforced epoxy resin composite plates. Furthermore, the flexibility matrix and stiffness matrix of the carbon-fiber-reinforced epoxy resin composite panels with an orthogonal braided structure can be obtained by correspondingly superimposing the flexibility matrix and stiffness matrix of two unidirectional carbon-fiber-reinforced epoxy resin composite panels. The flexibility matrix can represent the unidirectional laminate when the carbon fiber is 0°, while the unidirectional laminate at 90° can be obtained by the coordinate transformation matrix. Therefore, the direction cosine of the angle between the local coordinate and the integral coordinate at 90° can be obtained as follows:

(17) $$\begin{array}{l} l_{1}=\cos\left(x_{1},x\right)=0,m_{1}=\cos\left(x_{1},y\right)=1\\ l_{2}=\cos\left(y_{1},x\right)=-1,m_{2}=\cos\left(y_{1},y\right)=0 \end{array}$$

Then, the stress transformation matrix ${\left({\left[T_{ij}\right]}_{c}\right)}_{90}$ can be expressed as:

(18) $${\left({\left[T_{ij}\right]}_{c}\right)}_{90}=\left[\begin{array}{ccc} 0 & 1 & 0\\ 1 & 0 & 0\\ 0 & 0 & -1 \end{array}\right]$$

In the global coordinate system, the stress–strain relationship at any point in the laminates and the stiffness matrix $\left[{\left(C_{ij}^{G}\right)}_{k}\right]$ of the *K* layer unidirectional carbon fiber composite can be expressed as:

(19) $${\left\{\sigma_{i}\right\}}^{\left(G\right)}=\left[C_{ij}^{G}\right]{\left\{\varepsilon_{j}\right\}}^{\left(G\right)}$$

(20) $$\left[{\left(C_{ij}^{G}\right)}_{k}\right]={\left({\left[T_{ij}\right]}_{c}\right)}_{k}{\left({\left[S_{ij}\right]}_{k}\right)}^{-1}{\left({\left[T_{ij}\right]}_{c}^{T}\right)}_{k}$$

Therefore, the carbon fiber stiffness matrix ${\left[C_{ij}^{G}\right]}_{0}$ at 0° and the stiffness matrix ${\left[C_{ij}^{G}\right]}_{90}$ at 90° can be expressed as:

(21) $${\left[C_{ij}^{G}\right]}_{0}={\left[S_{ij}\right]}^{-1}=\left[\begin{array}{ccc} \frac{E_{11}^{2}}{E_{11}-E_{22}\nu_{12}^{2}} & \frac{E_{11}E_{22}\nu_{12}}{E_{11}-E_{22}\nu_{12}^{2}} & 0\\ \frac{E_{11}E_{22}\nu_{12}}{E_{11}-E_{22}\nu_{12}^{2}} & \frac{E_{11}E_{22}}{E_{11}-E_{22}\nu_{12}^{2}} & 0\\ 0 & 0 & \frac{E_{11}G_{12}}{E_{11}-E_{22}\nu_{12}^{2}} \end{array}\right]$$

(22) $${\left[C_{ij}^{G}\right]}_{90}=\left[\begin{array}{ccc} \frac{E_{11}E_{22}}{E_{11}-E_{22}\nu_{12}^{2}} & \frac{E_{11}E_{22}\nu_{12}}{E_{11}-E_{22}\nu_{12}^{2}} & 0\\ \frac{E_{11}E_{22}\nu_{12}}{E_{11}-E_{22}\nu_{12}^{2}} & \frac{E_{11}^{2}}{E_{11}-E_{22}\nu_{12}^{2}} & 0\\ 0 & 0 & \frac{E_{11}G_{12}}{E_{11}-E_{22}\nu_{12}^{2}} \end{array}\right]$$

The stiffness matrix ${\left[C_{ij}^{G}\right]}_{ORTHOG}$ of the orthogonal fiber laminates can be expressed as the superposition of two unidirectional fiber laminates:

(23) $${\left[C_{ij}^{G}\right]}_{ORTHOG}=\left[\begin{array}{ccc} \frac{E_{11}^{2}+E_{11}E_{22}}{E_{11}-E_{22}\nu_{12}^{2}} & \frac{2E_{11}E_{22}\nu_{12}}{E_{11}-E_{22}\nu_{12}^{2}} & 0\\ \frac{2E_{11}E_{22}\nu_{12}}{E_{11}-E_{22}\nu_{12}^{2}} & \frac{E_{11}^{2}+E_{11}E_{22}}{E_{11}-E_{22}\nu_{12}^{2}} & 0\\ 0 & 0 & \frac{2E_{11}G_{12}}{E_{11}-E_{22}\nu_{12}^{2}} \end{array}\right]$$

According to the classical laminate theory, when the laminate is symmetrical, the coupling stiffness is 0. Then, the tensile stiffness $Q_{ij}^{I}$ and bending stiffness $Q_{ij}^{III}$ of the laminate are expressed as:

(24) $$\begin{array}{l} Q_{ij}^{I}=\sum_{k=1}^{n}{\left(C_{ij}^{G}\right)}_{k}\left(z_{k}-z_{k-1}\right)\\ Q_{ij}^{\mathrm{III}}=\frac{1}{3}\sum_{k=1}^{n}{\left(C_{ij}^{G}\right)}_{k}\left(z_{{}_{k}}^{3}-z_{{}_{k-1}}^{3}\right) \end{array}$$

Therefore, when the thickness of the carbon fiber laminate is t, $z_{0}=-t/2$, $z_{1}=t/2$ and h=t. The tensile stiffness ${\left(Q_{ij}^{I}\right)}_{ORTHOG}$ and bending stiffness ${\left(Q_{ij}^{\mathrm{III}}\right)}_{ORTHOG}$ of the orthogonal carbon fiber laminate can be obtained by substituting the above formula:

(25) $$\begin{array}{l} {\left(Q_{ij}^{I}\right)}_{ORTHOG}=t\left[\begin{array}{ccc} \frac{E_{11}^{2}+E_{11}E_{22}}{E_{11}-E_{22}\nu_{12}^{2}} & \frac{2E_{11}E_{22}\nu_{12}}{E_{11}-E_{22}\nu_{12}^{2}} & 0\\ \frac{2E_{11}E_{22}\nu_{12}}{E_{11}-E_{22}\nu_{12}^{2}} & \frac{E_{11}^{2}+E_{11}E_{22}}{E_{11}-E_{22}\nu_{12}^{2}} & 0\\ 0 & 0 & \frac{2E_{11}G_{12}}{E_{11}-E_{22}\nu_{12}^{2}} \end{array}\right]\\ {\left(Q_{ij}^{\mathrm{III}}\right)}_{ORTHOG}=\frac{t^{3}}{12}\left[\begin{array}{ccc} \frac{E_{11}^{2}+E_{11}E_{22}}{E_{11}-E_{22}\nu_{12}^{2}} & \frac{2E_{11}E_{22}\nu_{12}}{E_{11}-E_{22}\nu_{12}^{2}} & 0\\ \frac{2E_{11}E_{22}\nu_{12}}{E_{11}-E_{22}\nu_{12}^{2}} & \frac{E_{11}^{2}+E_{11}E_{22}}{E_{11}-E_{22}\nu_{12}^{2}} & 0\\ 0 & 0 & \frac{2E_{11}G_{12}}{E_{11}-E_{22}\nu_{12}^{2}} \end{array}\right] \end{array}$$

The carbon fiber laminate is composed of carbon fiber and epoxy resin. It is necessary to establish a cooperative relationship between the carbon fiber and the epoxy resin to obtain the axial modulus, the transverse modulus, Poisson’s ratio, and the shear modulus of the laminate. In this study, fibers and resins are linked by a bridging matrix.

It is assumed that the interface between the fiber and the resin is ideal. Therefore, the mean internal stress of the fiber and the resin must be connected by a non-singular matrix.

(26) $$\left\{\sigma_{i}^{m}\right\}=\left[A_{ij}\right]\left\{\sigma_{i}^{f}\right\}$$

In this case, the fiber stress $\left\{\sigma_{i}^{f}\right\}$ in the laminate can be expressed as the integral stress $\left\{\sigma_{i}\right\}$:

(27) $$\left\{\sigma_{i}^{f}\right\}={\left(V_{f}\left[I\right]+V_{m}{\left[A_{ij}\right]}^{-1}\right)}^{-1}\left\{\sigma_{i}\right\}=\left[B_{ij}\right]\left\{\sigma_{i}\right\}$$

where $V_{f}$ and $V_{m}$ are the volume fractions of the carbon fiber and resin matrix in the fiber laminates, respectively. The matrix $\left[B_{ij}\right]$ is the stress distribution matrix of the fiber, and $\left[A_{ij}\right]\cdot \left[B_{ij}\right]$ is the stress distribution matrix of the matrix. The expression of the bridging matrix $\left[A_{ij}\right]$ can be obtained by substituting the formula into the mean-averaged constitutive equation of the fiber and the matrix:

(28) $$\left[A_{ij}\right]=V_{f}{\left(\left[S_{ij}\right]-\left[S_{ij}^{m}\right]\right)}^{-1}\left(\left[S_{ij}^{f}\right]-\left[S_{ij}\right]\right)/V_{m}$$

In the elastic stage, the flexibility matrix of the fiber, matrix, and unidirectional composite $\left[S_{ij}^{f}\right]$, $\left[S_{ij}^{m}\right]$, $\left[S_{ij}\right]$ can be expressed as a block diagonal matrix. The corresponding bridge matrix can also be expressed in the corresponding block diagonal form:

(29) $$\left[A_{ij}\right]=\left[\begin{array}{cc} {\left[A_{ij}\right]}_{\sigma } & 0\\ 0 & {\left[A_{ij}\right]}_{\tau } \end{array}\right]$$

where

(30) $$\begin{array}{l} {\left[A_{ij}\right]}_{\sigma }=V_{f}{\left({\left[S_{ij}\right]}_{\sigma }-{\left[S_{ij}^{m}\right]}_{\sigma }\right)}^{-1}\left({\left[S_{ij}^{f}\right]}_{\sigma }-{\left[S_{ij}\right]}_{\sigma }\right)/V_{m}\\ {\left[A_{ij}\right]}_{\tau }=V_{f}{\left({\left[S_{ij}\right]}_{\tau }-{\left[S_{ij}^{m}\right]}_{\tau }\right)}^{-1}\left({\left[S_{ij}^{f}\right]}_{\tau }-{\left[S_{ij}\right]}_{\tau }\right)/V_{m} \end{array}$$

Since it is in the elastic stage, ${\left[A_{ij}\right]}_{\tau }$ is a diagonal matrix, so only 6 elements in ${\left[A_{ij}\right]}_{\sigma }$ need to be determined. Since it is a unidirectional composite, the elements in the flexibility matrix have the following relations.

(31) $$S_{44}=1/G_{23}=2\left(1+\nu_{23}\right)/E_{22}=2\left(S_{22}-S_{23}\right)$$

Therefore, there are only five elements to be obtained: $A_{11},A_{22}=A_{33},A_{21},A_{32},A_{55}=A_{66}$. The bridging matrix ${\left[A_{ij}\right]}_{BM}$ is as follows:

(32) $${\left[A_{ij}\right]}_{BM}=\left[a_{ij}\right]=\left[\begin{array}{cccccc} a_{11} & a_{12} & a_{13} & 0 & 0 & 0\\ 0 & a_{22} & 0 & 0 & 0 & 0\\ 0 & 0 & a_{33} & 0 & 0 & 0\\ 0 & 0 & 0 & a_{44} & 0 & 0\\ 0 & 0 & 0 & 0 & a_{55} & 0\\ 0 & 0 & 0 & 0 & 0 & a_{66} \end{array}\right]$$

(33) $$\begin{array}{l} a_{11}=E^{m}/E_{11}^{f}\\ a_{22}=a_{33}=a_{44}=\beta +\left(1-\beta \right)\frac{E^{m}}{E_{22}^{f}}\left(0<\beta <1,\beta \;\mathrm{is}\;\mathrm{generally}\;0.3\right)\\ a_{55}=a_{66}=\alpha +\left(1-\alpha \right)\frac{G^{m}}{G_{12}^{f}}\left(0<\alpha <1,\alpha \;\mathrm{is}\;\mathrm{generally}\;0.3\right)\\ a_{12}=a_{13}=\frac{S_{12}^{f}-S_{12}^{m}}{S_{11}^{f}-S_{11}^{m}}\left(a_{11}-a_{22}\right)=\frac{E_{11}^{f}\nu^{m}-E^{m}\nu_{12}^{f}}{E_{11}^{f}-E^{m}}\left(a_{22}-a_{11}\right) \end{array}$$

where $E_{m}$, $G_{m}$, and $\nu_{m}$ are the elastic modulus, the shear modulus, and Poisson’s ratio of the matrix, respectively. $E_{11}^{f}$, $E_{22}^{f}$, $G_{12}^{f}$, and $\nu_{12}^{f}$ are the axial modulus, transverse modulus, shear modulus, and Poisson’s ratio of the fiber, respectively. α and β are bridge parameters.

The axial modulus, axial Poisson’s ratio, transverse modulus, and transverse shear modulus of the unidirectional composite can be obtained as follows:

(34) $$\begin{array}{l} E_{11}=V_{f}E_{11}^{f}+V_{m}E^{m}\\ \nu_{12}=V_{f}\nu_{12}^{f}+V_{m}\nu^{m}\\ E_{22}=\frac{\left(V_{f}+V_{m}a_{11}\right)\left(V_{f}+V_{m}a_{22}\right)}{\left(V_{f}+V_{m}a_{11}\right)\left(V_{f}S_{22}^{f}+a_{22}V_{m}S_{22}^{m}\right)+V_{f}V_{m}\left(S_{21}^{m}-S_{21}^{f}\right)a_{12}}\\ G_{12}=\frac{G_{12}^{f}G^{m}\left(V_{f}+V_{m}a_{66}\right)}{V_{f}G_{m}+V_{m}G_{12}^{f}a_{66}}\\ G_{23}=\frac{G_{23}^{f}G^{m}\left(V_{f}+V_{m}a_{22}\right)}{V_{f}G^{m}+V_{m}G_{23}^{f}a_{22}} \end{array}$$

Therefore, the bending moment and stress of the laminates can be calculated according to the basic performance parameters of the fiber and the matrix and the bending deflection of the laminates.

This theory is based on a unidirectional composite. Therefore, the selected data are the performance parameters and volume content of the fiber and resin matrix in the unidirectional composite. After the stiffness matrix and basic performance parameters of the carbon fiber composite were determined, the strain during bending was also analyzed. The classical laminate theory is based on Kirchhoff’s basic assumption in the theory of elastic thin sheets, which consists of the following two premises:

(1) The plane strain assumption: the strain of the laminates in the out-of-plane direction is much smaller than in the other two directions. Therefore, the strain is ignored.
(2) The straight normal assumption: The displacement in the plane varies linearly along the radial direction of the laminate. Normal lines perpendicular to the central plane remain straight after deformation.

For the unidirectional laminate, the fiber direction is used as the *X*-axis, the direction perpendicular to the fiber inside the plane is used as the *Y*-axis, and the direction perpendicular to the plane is used as the *Z*-axis. The displacement of a point in the laminate in the *X*, *Y*, and *Z* directions is denoted by the displacement on the coordinate axis u=u(x,y,z), v=v(x,y,z), w=w(x,y,z).

According to assumption (1), the deflection of the laminates, namely, the displacement component in the *Z* direction, is independent of the *Z* coordinate. According to assumption (2), the displacement components along the *X* and *Y* directions in the laminate plane are linear functions of the *Z* coordinates. Therefore, the displacement components along the coordinate axis are expressed as follows:

(35) $$\begin{array}{l} u=u\left(x,y,z\right)=u^{0}\left(x,y\right)+zF_{1}\left(x,y\right)\\ v=v\left(x,y,z\right)=v^{0}\left(x,y\right)+zF_{2}\left(x,y\right)\\ w=w\left(x,y,z\right)=w^{0}\left(x,y\right) \end{array}$$

where *u*^0^, *v*^0^, and *w*^0^ are the displacement components of the middle plane of the laminate along the *X*, *Y*, and *Z* directions, respectively. *F*_1_ and *F*_2_ are functions of *x* and *y*, respectively.

It can be obtained from the strain geometric equation that the normal strain $\varepsilon_{xx}$, $\varepsilon_{yy}$, $\varepsilon_{zz}$ and shear strain $\varepsilon_{xy}$, $\varepsilon_{xz}$, $\varepsilon_{yz}$ of the laminates are expressed as follows:

(36) $$\begin{array}{l} \varepsilon_{xx}=\frac{\partial u}{\partial x},\varepsilon_{yy}=\frac{\partial v}{\partial y},\varepsilon_{zz}=\frac{\partial w}{\partial z}\\ \varepsilon_{xy}=\frac{1}{2}\left(\frac{\partial u}{\partial y}+\frac{\partial v}{\partial x}\right),\varepsilon_{xz}=\frac{1}{2}\left(\frac{\partial u}{\partial z}+\frac{\partial w}{\partial x}\right),\varepsilon_{yz}=\frac{1}{2}\left(\frac{\partial v}{\partial z}+\frac{\partial w}{\partial y}\right) \end{array}$$

By combining the displacement component function with the strain equation, the out-of-plane shear strain $\gamma_{xz}$ and $\gamma_{yz}$ of the laminates can be obtained:

(37) $$\begin{array}{l} \gamma_{xz}=2\varepsilon_{xz}=\left(\frac{\partial u}{\partial z}+\frac{\partial w}{\partial x}\right)=F_{1}\left(x,y\right)+\frac{\partial w^{0}}{\partial x}\\ \gamma_{yz}=2\varepsilon_{yz}=\left(\frac{\partial v}{\partial z}+\frac{\partial w}{\partial y}\right)=F_{2}\left(x,y\right)+\frac{\partial w^{0}}{\partial y} \end{array}$$

According to the assumption of small deformation and plane strain, it can be deduced that:

(38) $$F_{1}\left(x,y\right)=-\frac{\partial w^{0}}{\partial x},F_{2}\left(x,y\right)=-\frac{\partial w^{0}}{\partial y}$$

Therefore, the geometric equation at any point on the laminates can be expressed as follows:

(39) $$\begin{array}{l} \varepsilon_{xx}=\frac{\partial u^{0}}{\partial x}-z\frac{\partial^{2}w^{0}}{\partial x^{2}}=\varepsilon_{xx}^{0}+z\kappa_{xx}^{0}\\ \varepsilon_{yy}=\frac{\partial v^{0}}{\partial y}-z\frac{\partial^{2}w^{0}}{\partial y^{2}}=\varepsilon_{yy}^{0}+z\kappa_{yy}^{0}\\ \gamma_{xy}=2\varepsilon_{xy}=\left(\frac{\partial u^{0}}{\partial y}+\frac{\partial v^{0}}{\partial x}\right)-2z\frac{\partial^{2}w^{0}}{\partial x\partial y}=2\varepsilon_{xy}^{0}+2z\kappa_{xy}^{0} \end{array}$$

In the formula, $\varepsilon_{xx}$, $\varepsilon_{yy}$, $\gamma_{xy}$ are the strains of the laminates. $\varepsilon_{xx}^{0}$, $\varepsilon_{yy}^{0}$, $\varepsilon_{xy}^{0}$ are the middle plane strains of the laminates. $\kappa_{xx}^{0}$, $\kappa_{yy}^{0}$ represent the middle-plane curvature of the laminates. $\kappa_{xy}^{0}$ is the torsion rate of the middle plane of the laminate. Any point on the laminates can be expressed by the strain, curvature, and torsion of the middle plane. In this paper, it was assumed that the failure of FMLs occurs when the strain of the fiber layer reaches the elongation limit. Considering that the aluminum alloy in the FMLs has good plastic deformation ability, it has a certain promotion effect on the elongation deformation of the carbon fiber. However, as the bond interface between the carbon fiber and aluminum alloy was weak, when the number of laminates increased, the promotion effect would gradually weaken until the final failure. Therefore, the elongation correction coefficient was introduced to obtain the elongation limit of the different layers.

(40) $$dl=m\cdot dl_{0}=1.5^{3/n^{2}}dl_{0}$$

where $dl_{0}$ is the initial elongation of the fiber, dl is the modified elongation, *m* is the correction coefficient, and *n* is the number of fiber layers in the laminate.

The bending deformation of the carbon fiber composites under bending stress is a slow process. The bending process can be regarded as quasi-static. Combined with the stiffness of the carbon fiber composites obtained above and the strain relation formula obtained in this section, a stress balance analysis of carbon fiber composites under bending stress is carried out in this study.

The internal force and internal torque applied to the laminates are expressed as $N_{xx}$, $N_{yy}$, $N_{xy}$, $M_{xx}$, $M_{yy}$, and $M_{xy}$. The internal force and internal moment are balanced with the stress on the section:

(41) $$\left\{\begin{array}{c} N_{xx}\\ N_{yy}\\ N_{xy} \end{array}\right\}=\int_{-h/2}^{h/2}\left\{\begin{array}{c} \sigma_{xx}\\ \sigma_{yy}\\ \sigma_{xy} \end{array}\right\}dz=Q_{ij}^{I}\left\{\begin{array}{c} \varepsilon_{xx}^{0}\\ \varepsilon_{yy}^{0}\\ 2\varepsilon_{xy}^{0} \end{array}\right\}+Q_{ij}^{\mathrm{II}}\left\{\begin{array}{c} \kappa_{xx}^{0}\\ \kappa_{yy}^{0}\\ 2\kappa_{xy}^{0} \end{array}\right\}$$

(42) $$\left\{\begin{array}{c} M_{xx}\\ M_{yy}\\ M_{xy} \end{array}\right\}=\int_{-h/2}^{h/2}\left\{\begin{array}{c} \sigma_{xx}\\ \sigma_{yy}\\ \sigma_{xy} \end{array}\right\}zdz=Q_{ij}^{\mathrm{II}}\left\{\begin{array}{c} \varepsilon_{xx}^{0}\\ \varepsilon_{yy}^{0}\\ 2\varepsilon_{xy}^{0} \end{array}\right\}+Q_{ij}^{\mathrm{III}}\left\{\begin{array}{c} \kappa_{xx}^{0}\\ \kappa_{yy}^{0}\\ 2\kappa_{xy}^{0} \end{array}\right\}$$

where

(43) $$h=\sum_{k=1}^{N}\left(z_{k+1}-z_{k}\right)$$

The above formula shows the thickness of the laminates. $z_{k}$ and $z_{k+1}$ are the coordinates of the upper and lower top surfaces of layer *K*.

Therefore, the stiffness matrix can be expressed in blocks.

(44) $$\left\{\begin{array}{c} N\\ M \end{array}\right\}=\left[\begin{array}{cc} Q^{I} & Q^{\mathrm{II}}\\ Q^{\mathrm{II}} & Q^{\mathrm{III}} \end{array}\right]\left\{\begin{array}{c} \varepsilon^{0}\\ \kappa^{0} \end{array}\right\}$$

Based on the above analysis, it can be determined that, when the laminate only bears the bending stress, its bending moment can be obtained:

(45) $$\left\{\begin{array}{c} M_{xx}\\ M_{yy}\\ M_{xy} \end{array}\right\}=Q_{ij}^{\mathrm{III}}\left\{\begin{array}{c} \kappa_{xx}^{0}\\ \kappa_{yy}^{0}\\ 2\kappa_{xy}^{0} \end{array}\right\}=\frac{t^{3}}{12}\left[\begin{array}{ccc} \frac{E_{11}^{2}+E_{11}E_{22}}{E_{11}-E_{22}\nu_{12}^{2}} & \frac{2E_{11}E_{22}\nu_{12}}{E_{11}-E_{22}\nu_{12}^{2}} & 0\\ \frac{2E_{11}E_{22}\nu_{12}}{E_{11}-E_{22}\nu_{12}^{2}} & \frac{E_{11}^{2}+E_{11}E_{22}}{E_{11}-E_{22}\nu_{12}^{2}} & 0\\ 0 & 0 & \frac{2E_{11}G_{12}}{E_{11}-E_{22}\nu_{12}^{2}} \end{array}\right]\left\{\begin{array}{c} \kappa_{xx}^{0}\\ \kappa_{yy}^{0}\\ 2\kappa_{xy}^{0} \end{array}\right\}$$

The laminate bears the pure bending moment $M_{xx}$. The middle plane curvature $\kappa_{xx}^{0}$ can be obtained from geometric conditions.

### 3.3. Laminate Theory

In Section 3.1 and Section 3.2, the bending deformation behavior of the aluminum alloy layer and carbon fiber layer was studied. CARALL is made of an alternately stacked aluminum alloy and carbon fiber, and the outer layer comprises aluminum alloy. Therefore, the bending moment and bending stress of the whole CARALL during bending deformation can be expressed as follows:

(46) $$M_{n+1/n}=\{\begin{array}{c} \sum_{1}^{j=n/2}\left(M_{ij}+M_{oj}\right)+\sum_{1}^{k=n}M_{ck}+M_{m}\;\;n\;\mathrm{is}\;\mathrm{even}\\ \sum_{1}^{j=\left(n+1\right)/2}\left(M_{ij}+M_{oj}\right)+\sum_{1}^{k=n}M_{ck}\;\;\;n\;\mathrm{is}\;\mathrm{odd} \end{array}$$

(47) $$\sigma_{n+1/n}=\frac{M_{n+1/n}}{b{\left[\left(n+1\right)t_{al}+nt_{c}\right]}^{2}}$$

where n is the number of carbon fiber layers in the CARALL. $M_{ij}$, $M_{m}$, and $M_{oj}$ are the bending moment of the aluminum alloy layers. $M_{ck}$ is the bending moment of the carbon fiber layers. b is the width of the laminates. $t_{al}$ and $t_{c}$ are the thickness of the single aluminum alloy and the carbon fiber layer, respectively.

It can be deduced from the above formula that the laminate’s bending stress state is related to its bending radius and thickness. The thickness of the laminate is determined by the thickness of the aluminum plate and the thickness of the carbon fiber layer.

In this paper, studies are carried out on laminates in 3 lay-ups, namely 2/1, 3/2, and 4/3. The specific calculation methods are as follows.

(1) Bending theory of 2/1 CARALL

The 2/1 CARALL is made by sandwiching a carbon fiber composite between 2 aluminum alloy layers. Through the stress analysis, it can be observed that the outer aluminum alloy layer experiences tensile deformation during bending. In comparison, the inner aluminum alloy layer is compressed during bending.

**Figure 7:**
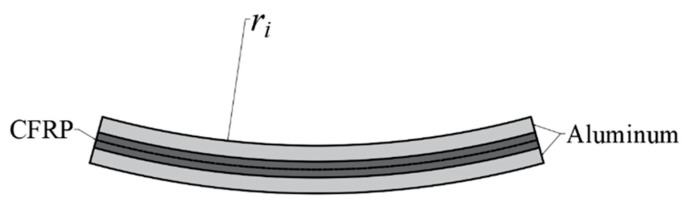
Bending diagram of 2/1 CARALL.

The elongation of the carbon fiber used in this study is 1.0%. When the elongation of the carbon fiber reaches its limit, the carbon fiber layer breaks and the laminate lose its bearing capacity. Considering the excellent plasticity of the aluminum alloy in CARALLs, the ultimate elongation of the carbon fiber is improved to 3.375% according to Formula (40). A bending diagram for 2/1 CARALL is shown in Figure 7, where the radius of the neutral layer is $r_{i}+t_{al}+t_{c}/2$.

According to the geometric analysis of bending deformation, the maximum elongation of the carbon fiber in the 2/1 CARALL is located on the upper or lower surfaces of the carbon fiber layer. This assumes that the neutral bending surface of the carbon fiber composite always coincides with the middle plane of the carbon fiber layer in the bending process. The total bending moment of the CARALL is expressed as:

(48) $$M_{2/1}=M_{i}+M_{c}+M_{o}$$

where $M_{i}$ and $M_{o}$ are the bending moments of the inner and outer aluminum alloy, respectively. $M_{c}$ is the bending moment of the middle carbon fiber layer. The whole laminate only bears a pure bending moment. The bending stress of the 2/1 CARALL can be expressed as:

(49) $$\sigma_{2/1}=\frac{My}{I_{z}}=\frac{6M_{2/1}}{b{\left(2t_{al}+t_{c}\right)}^{2}}$$

(2) Bending theory of 3/2 CARALL

**Figure 8:**
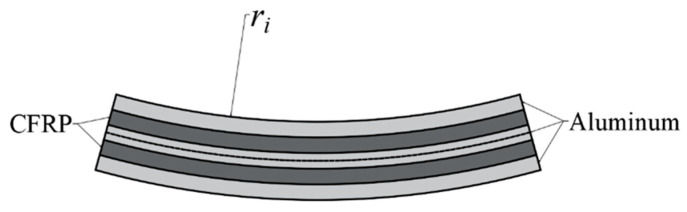
Bending diagram of 3/2 CARALL.

The 3/2 CARALL is made by sandwiching 2 carbon fiber layers with 3 aluminum alloy layers. According to the stress analysis, it was determined that the outer aluminum alloy layer undergoes tensile deformation, the inner aluminum alloy layer undergoes compression deformation, and the middle aluminum alloy layer undergoes complex deformation, including both tensile and compression. Therefore, the aluminum alloy is considered to be stratified when calculating the bending moment during bending. In 3/2 laminates, the presence of an aluminum alloy can improve the elongation of the carbon fiber. However, as the number of layers increases, the increasingly weak bonding surfaces in the laminates limit elongation improvement. According to Formula (40), the elongation is revised to 1.355%. A bending diagram for the 3/2 CARALL is shown in Figure 8, where the radius of the neutral layer is $r_{i}+3t_{al}/2+t_{c}$.

According to the geometric analysis of bending deformation, the maximum elongation of the carbon fiber of the 3/2 CARALL is located on the lower surface of the outer carbon fiber. The bending moment of the 3/2 CARALL is:

(50) $$M_{3/2}=M_{i}+M_{c1}+M_{m}+M_{c2}+M_{o}$$

where $M_{i}$, $M_{m}$, and $M_{o}$ are the bending moments of the inner, middle, and outer aluminum alloy layers, respectively. $M_{c1}$ and $M_{c2}$ are the bending moments of the carbon fiber layers. The bending stress of 3/2 CARALL can be expressed as:

(51) $$\sigma_{3/2}=\frac{My}{I_{z}}=\frac{6M_{3/2}}{b{\left(3t_{al}+2t_{c}\right)}^{2}}$$

(3) Bending theory of 4/3 CARALL

**Figure 9:**
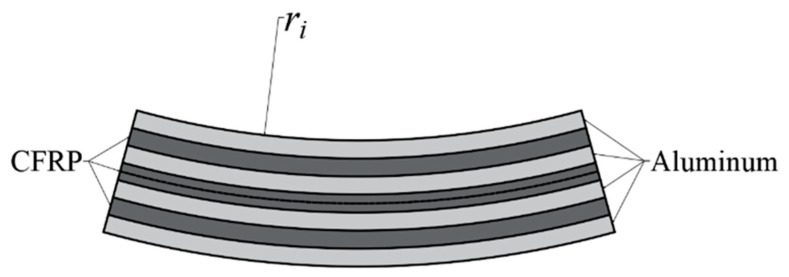
Bending diagram of 4/3 CARALL.

The 3/4 CARALL is made of 4 aluminum alloy layers sandwiched with 3 carbon fiber layers. By conducting a stress analysis, it was found that the outer two aluminum alloy layers experience tensile deformation, and the inner two aluminum alloy layers experience compression deformation. In the 4/3 CARALL, there are more weak bonding surfaces between the carbon fiber and aluminum alloy. According to Formula (40), the elongation of the carbon fiber is modified to 1.145%. A bending diagram of the 4/3 CARALL is shown in Figure 9, where the radius of the neutral layer is $r_{i}+2t_{al}+3t_{c}/2$.

According to the geometric analysis of bending deformation, the maximal elongation of the carbon fiber in the 4/3 CARALL occurs on the lower surface of the inner carbon fiber. The bending moment and bending stress of the 4/3 CARALL are as follows:

(52) $$M_{4/3}=M_{i_{1}}+M_{c1}+M_{i_{2}}+M_{c2}+M_{o_{2}}+M_{c3}+M_{o_{1}}$$

(53) $$\sigma_{4/3}=\frac{My}{I_{z}}=\frac{6M_{4/3}}{b{\left(4t_{al}+3t_{c}\right)}^{2}}$$

## 4. Results and Discussion

### 4.1. Experimental Results for Bending Strength

**Figure 10:**
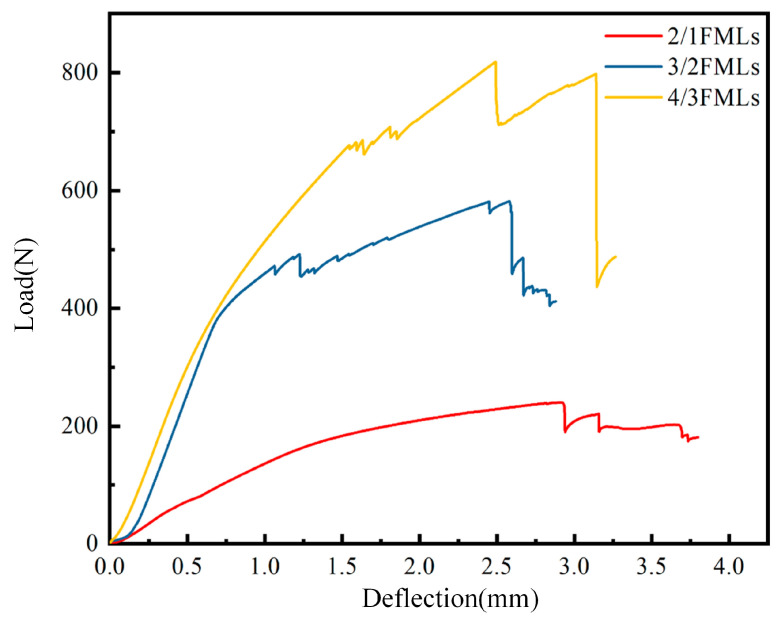
Load-deflection curves of CARALLs with different lay-ups.

The three-point bending test was carried out for three different layups of carbon fiber aluminum alloy laminates to obtain the load-deflection curves shown in Figure 10. For the convenience of comparison, only one set of results from repeated experiments is shown. As the three-point bending test was carried out, the carbon fiber aluminum alloy laminate gradually produced bending deformation, and the initial curve was linear. As the bending load gradually increased, the metal layer in the laminate started to deform plastically, and the slope of the curve became more gradual. When the load was further increased, the carbon fiber in the laminate gradually failed to fracture, and the load-deflection curve fluctuated. From Figure 10, it can be observed that the curve of the 4/3 laminate has the most fluctuations. Eventually, the laminate reached the bending load limit, and the test was finished.

**Figure 11:**
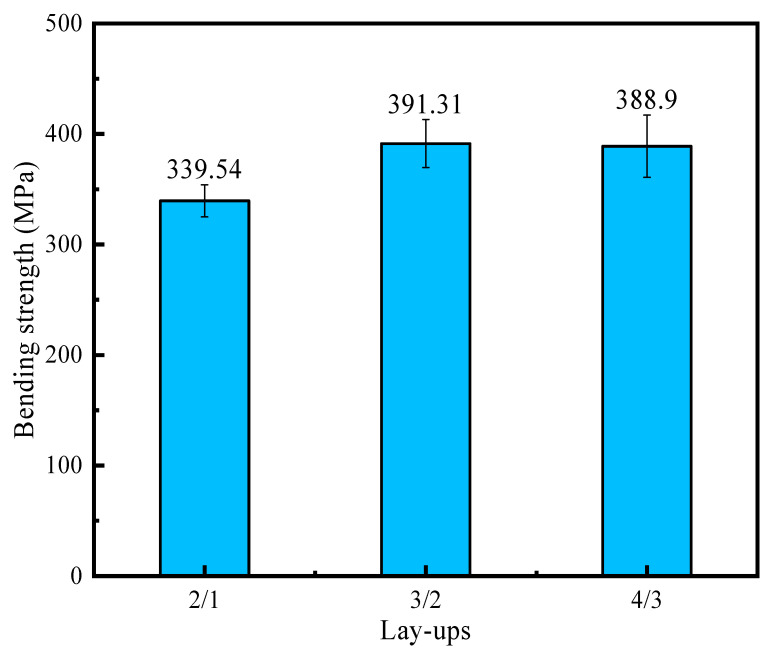
Comparison of the bending strength of laminates in three different lay-ups.

The test results for the bending strength of the 2/1, 3/2, and 4/3 laminates are 339.54 MPa, 391.31 MPa, and 388.9 MPa, respectively, and their standard deviations are 14.54 MPa, 21.75 MPa, and 28.08 MPa. No outliers occurred during the whole experiment. The bending strength of CARALLs with different lay-ups are shown in Figure 11. The bending strength of the CARALLs increases first and then decreases with the increasing number of layers. The highest strength occurs when the lay-up is 3/2. The error bar in Figure 11 represents the standard deviation of the bending test in different lay-ups. It can be seen that the standard deviation of the test results also increases with the increase in the number of layers, which indicates that the bending strength of the CARALLs becomes unstable.

**Figure 12:**
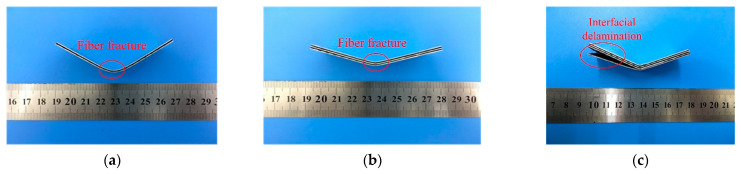
Failure modes of CARALLs in three different lay-ups: (**a**) 2/1 CARALL, (**b**) 3/2 CARALL, (**c**) 4/3 CARALL.

The bending samples of the CARALLs are shown in Figure 12. It can be observed that the major failure behavior of the CARALL with the 3/2 lay-up is a fiber fracture during the three-point bending test. When the laminates with the 4/3 lay-up are subjected to bending stress, the failure mainly comprises interfacial delamination.

**Figure 13:**
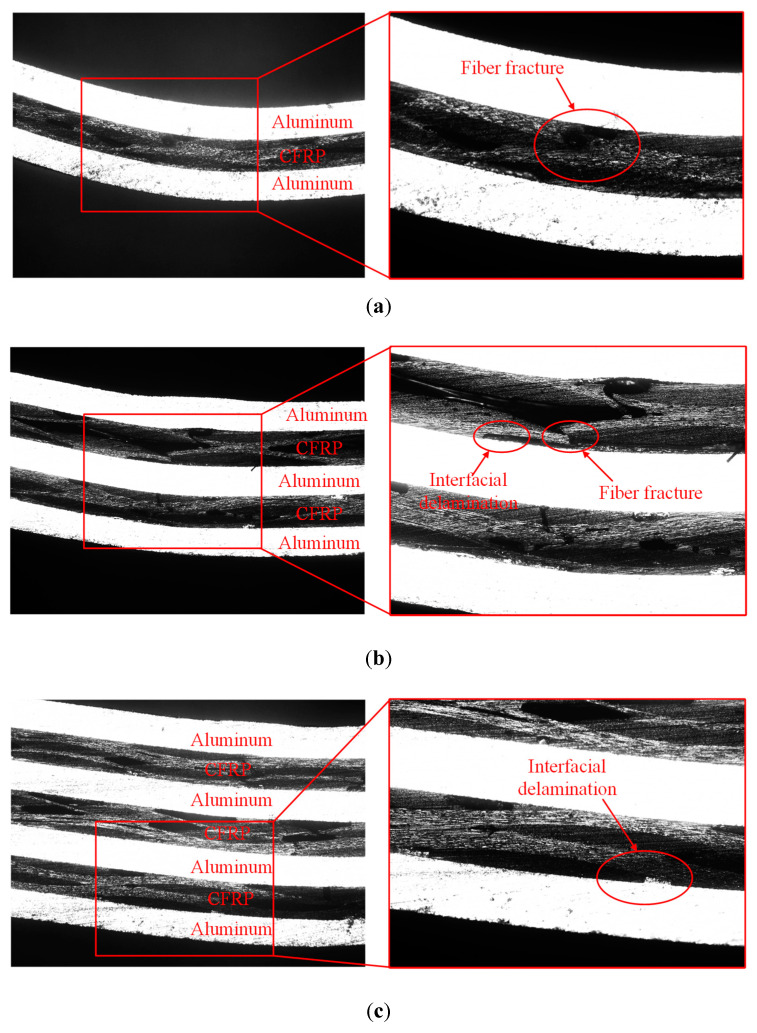
Micrograph of bending fractures with different lay-ups: (**a**) 2/1 CARALL, (**b**) 3/2 CARALL, (**c**) 4/3 CARALL.

Figure 13 shows different bending fractures in the 2/1, 3/2, and 4/3 CARALLs. The bending fracture mainly comprises the internal fiber fracture in the 2/1 CARALL. When the number of layers increases, the 3/2 CARALL exhibits both fiber fracture and a few interfacial delaminations. As for the 4/3 CARALL, the laminate shows only interfacial delamination.

It is essential to analyze the mechanisms of this phenomenon. The plastic ability differs significantly because of the difference between the carbon fiber and aluminum alloy. In the bending deformation, the excellent plasticity of the aluminum alloy can slightly improve the elongation of the carbon fiber. However, the difference in the plastic properties also causes the bonding surfaces of the aluminum alloy and fiber more significant stress. This interlaminar stress causes interfacial delamination between the carbon fiber and aluminum alloy. Although the volume content of carbon fibers increases with the increase in fiber layers, the number of interfaces between the carbon fiber and aluminum alloy also increases correspondingly. This increase in the number of carbon–aluminum interfaces weakens the strength provided by the carbon fiber content, resulting in the bending strength of the 4/3 CARALL being weaker than that of the 3/2 CARALL.

The bending test shows that simply increasing the number of layers and increasing the carbon fiber content does not cause an unlimited increase in the bending performance of the CARALL. When the number of layers reaches a certain level, the CARALL reaches its performance peak, i.e., in the 3/2 laminate.

### 4.2. Verification of the Modified Theoretical Bending Model

In this paper, a prediction theory of bending strength for CARALL is proposed. A specific strength prediction formula is given according to different laminates with different lay-ups. According to the theoretical calculations shown in Section 3.3, the bending strength of three kinds of CARALL can be obtained. Through the derivation process, the bending moment and stress of the laminate are calculated according to the material’s performance parameters given by the manufacturer, which are shown in Table 2. Then, the bending strength under the theoretical state is obtained. The results of the experimental test are also shown in Figure 11 in the previous step.

**Figure 14:**
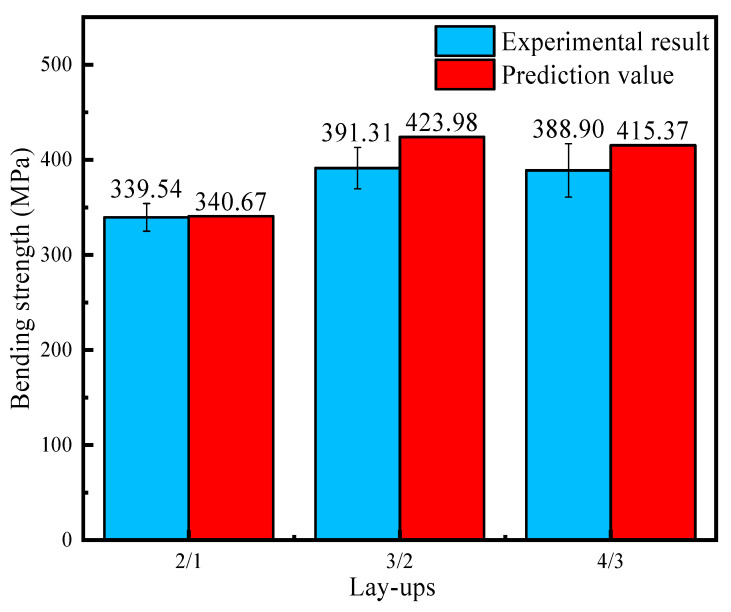
Comparison of the bending strength test results and theoretical predictions for three different lay-ups of CARALLs.

A comparison between the prediction value and the test results for bending strength is shown in Figure 14. It can be seen that the theoretical strength of the CARALLs increases first and then decreases with the increase in the number of layers. The bending strength of the CARALL reaches its highest level when the lay-up is 3/2, which is consistent with the bending test results.

**Figure 15:**
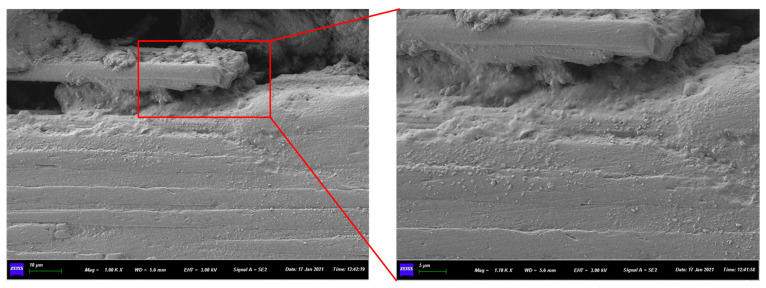
SEM test of CARALL.

By comparing the theoretical value of CARALLs with the experimental results, it is found that the bending strength calculated by making theoretical predictions is slightly higher than the experimental results for different lay-ups. Due to the influence of preparation technology, there are inevitably experimental errors, mainly in the carbon fiber prepreg and aluminum alloy bonding effect. During the lamination process, the laminate is extruded and vacuumed. However, a few pores still remain between the carbon fiber and aluminum alloy layers. Figure 15 shows a microscopic image of the CARALLs under a scanning electron microscope. Pores in the middle of the laminates can be clearly observed. As a result, the CARALL contains carbon fiber, resin, aluminum alloy, and pores. Therefore, the bending strength is lower than the ideal prediction during the bending experiment.

The theoretical model succeeded in analyzing the bending strength compared to the experimental results, and the prediction error was about 8.4%. Therefore, it is feasible to use this model to accurately predict the bending strength of the laminates.

### 4.3. Influencing Factors of Bending Deformation Behavior

It is assumed that, when the carbon fiber layer in the laminate reaches the elongation rate, the laminate reaches its bearing limit, and the bending strength of the laminate is calculated on this basis. Based on the previous theoretical research, this section studies the bending deformation behavior of laminates by analyzing the factors influencing laminates under different degrees of bending deformation.

**Figure 16:**
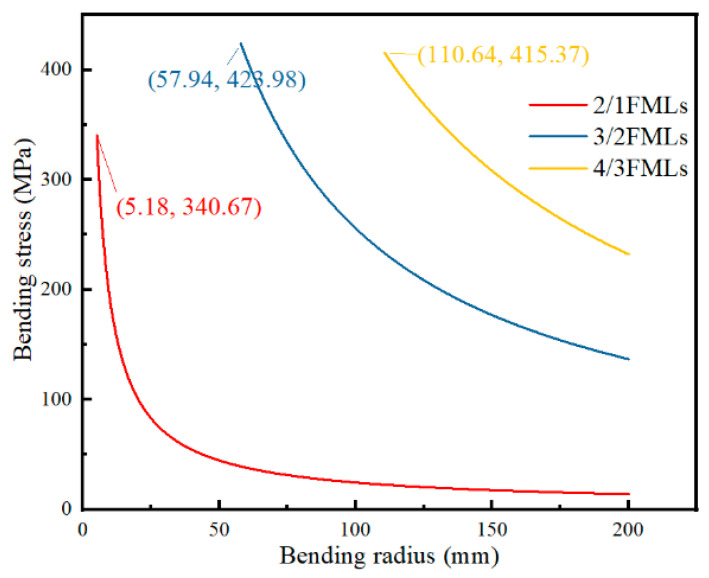
Bending radius–bending stress curves of CARALLs with different lay-ups.

The geometric analysis of the bending deformation of CARALLs shows that the bending radius of the laminates is infinite at the initial bending stage. With the increase in the degree of bending deformation, the bending radius gradually decreases. When the deformation limit of the laminates is reached, the bending radius of the laminates stops decreasing. The bending radius–bending stress curve obtained from the theoretical model of bending deformation is shown in Figure 16.

Figure 16 shows that the ultimate bending radius of the 2/1 laminate is the minimum, and the radius of the 4/3 laminate is the maximum. Since the difference between the three laminates is reflected in the different number of layers, the thickness of the 4/3 laminate is the maximum thickness. The ultimate bending radius is calculated according to the maximum elongation of the carbon fibers in the laminate; thus, it decreases with the increase in the number of layers.

According to the slope shown in Figure 16, the bending stress increases gradually with the decrease in the bending radius. The increasing amplitude of the bending stress is significant when the bending radius is closer to the minimum, especially when the lamination is 2/1. Because the limit bending radius of the 3/2 and 4/3 laminates is slightly larger, the further increase in the amplitude of the bending stress is limited. Therefore, it can be observed from the figure that the amplitude of the stress increase of the 2/1 laminate is higher than the 3/2 and 4/3 laminates.

**Figure 17:**
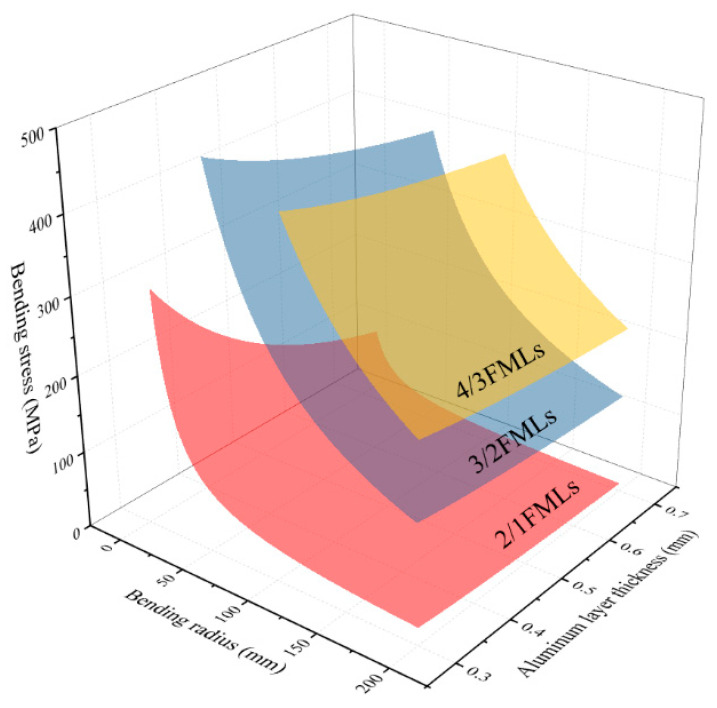
The bending stress curve varies with the bending radius of the laminates and the thickness of the aluminum layer under different lay-ups.

**Figure 18:**
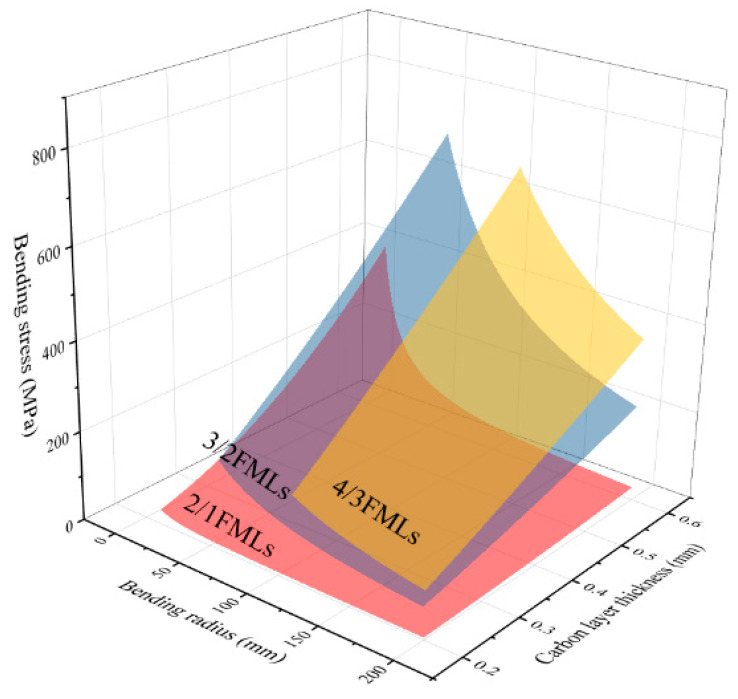
The bending stress curve varies with the bending radius of the laminates and the thickness of the carbon fiber layer under different lay-ups.

The influence of the thickness variation of the laminate’s component materials and the bending radius on the bending stress of the laminate was analyzed using the theoretical model of bending deformation. A three-dimensional figure is shown in Figure 17 and Figure 18. The above figure shows that the bending strength of the FMLs increases with the increase in the thickness of the carbon fiber layer, while the bending strength decreases with the increase in the thickness of the aluminum alloy layer. Through an analysis of the layer structure of CARALLs, the volume proportion of the carbon fiber materials in laminates increases with the increase in the carbon fiber thickness. The intensity of the carbon fiber is higher than that of the aluminum alloy. The carbon fiber is used as the main stress-bearing part in the laminate. Therefore, the laminates with greater fiber thickness have higher bending strength. Similarly, when the thickness of the aluminum alloy layer increases, the content of the weak aluminum alloy is higher, resulting in laminates with lower bending strength.

Analysis of the variation of the curves in Figure 17 and Figure 18 shows that the increasing trend of the bending strength gradually slows down as the thickness of the carbon fiber increases. When the thickness of the aluminum alloy layer increases, the decreasing trend of the bending strength of the laminate also slows down. The ideal case analysis shows that, when the thickness of the carbon fiber layers increases to infinity, the entire laminate is the carbon fiber laminate. The bending strength of the carbon fiber laminate is constant under the same preparation conditions. Therefore, with the increase in carbon fiber layer’s thickness, the increasing trend will gradually slow down and reach the carbon fiber’s bending strength. Similarly, when the thickness of the aluminum alloy layer increases to infinity, the entire laminate is an aluminum alloy sheet. This trend also gradually decreases and finally reaches the bending strength of the aluminum alloy.

After determining the aluminum layer, the main factors that affect the bending strength of the fiber metal laminate are the fiber content, the thickness of the carbon fiber layer, and the state of the layup.

**Figure 19:**
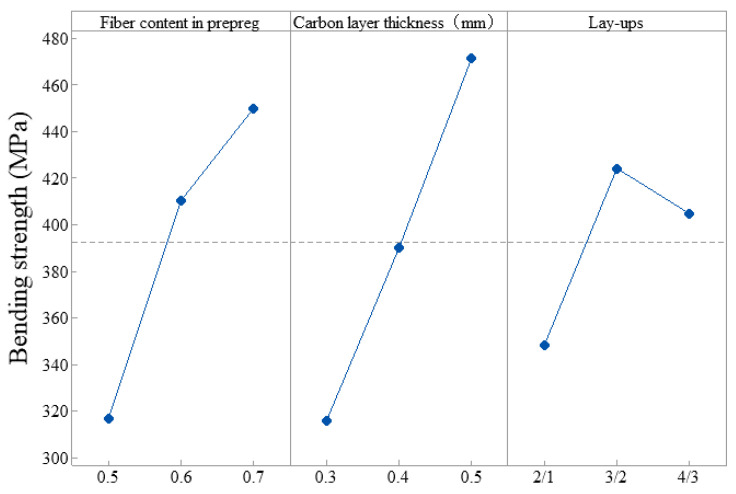
Main effect plot of means.

Based on the theory of bending deformation, Taguchi design analysis was used to obtain the influence of the fiber content of the prepreg, the fiber thickness, and the lay-ups on the bending strength of the CARALLs. The output response is shown in Table 3, and the main effect plot of means is shown in Figure 19. According to the row rank in the response table, the degree of influence of the process parameters on the bending strength of the CARALLs is as follows: the thickness of the carbon fiber layer, the fiber content of the prepreg, and the lay-ups. The influence of the carbon fiber is more significant, as the carbon fiber is the main bearing part of CARALLs. It can be seen from the main effect diagram that the increase in the thickness of the carbon layer and the fiber content of the prepreg is beneficial to the increase in the bending strength of the CARALLs. The properties of the 3/2 CARALL are the best.

## 5. Conclusions

This article focused on the bending deformation behavior of carbon fiber aluminum alloy laminates. According to different lay-ups of laminates, a theoretical model of bending deformation was established, and a three-point bending test was carried out. The bending test results and theoretical predictions were compared to verify the feasibility of the theoretical model. Finally, the factors influencing the bending deformation of laminates were investigated. The conclusions are as follows:

(1) The bending strength of CARALLs with different lay-ups was tested using a three-point bending test. Through comparison, it was found that the bending strength of the CARALLs increased first and then decreased with the increase in the number of layers. The maximum bending strength of the CARALLs was 391.31 MPa when the lay-up was 3/2. The standard deviation of the bending strength test increased with the increase in the number of layers, indicating that the greater the number of layers, the worse the stability of the bending performance of the CARALLs.
(2) The theoretical model was in good agreement with the test results. By comparing the results of the three-point bending test with the predictions of the theoretical model, it was found that the bending strength test results of the three kinds of CARALLs were 339.54, 391.31, and 388.90 MPa. The predictions of the model were 340.67, 423.98, and 415.37 MPa. The prediction error is therefore 8.4%. The variation trend of bending strength predicted by the model is consistent with the actual tests. Within the error range, it is feasible to predict the bending strength of CARALLs using the theoretical model established in this study.
(3) We analyzed the influence of process parameters, such as the laminate composition and bending radius, on the bending strength. The results showed that the bending stress of laminates increased as the bending radius decreased, especially for the 2/1 laminate. When the thickness of the carbon fiber layer increased, the bending strength of the laminates increased. The influence of the carbon fiber layer’s thickness on the bending strength of the CARALLs is more significant than the fiber content of the prepreg and the lay-ups.
(4) The mechanism of the variation in the bending strength of the fiber metal laminates was analyzed. During the preparation process, some pores were caused by bubbles in the CARALLs. Therefore, the test strength of the laminates was slightly lower than that obtained using theoretical predictions. The increase in the number of layers increased the volume content of the carbon fiber and the number of weak interfaces between the carbon fiber and the aluminum alloy. It resulted in the laminates under the 3/2 lay-up having the highest bending strength. As the thickness of the carbon layer increased, the volume fraction of carbon fiber in the laminates increased. Therefore, the strength of the laminates increased.

## Figures and Tables

**Table 1:** Properties of the carbon fiber.

Tensile Strength/GPa	Elastic Modulus/GPa	Elongation/%	Linear Density/g·cm^−1^	Diameter/μm
3	230	1.0	1.78	7

**Table 2:** Primary performance parameters of the unidirectional composites.

Material	$E_{11}$/GPa	$\nu_{12}$	$E_{22}$/GPa	$G_{12}$/GPa	$G_{23}$/GPa	$\nu_{23}$
Fiber	230	0.2	15	15	7	0.07
Resin	3.2	0.35	3.2	1.3	1.3	0.35

**Table 3:** Average response table of process parameters.

Levels	Fiber Content of Prepreg	Carbon Layer Thickness	Lay-Ups
1	316.9	315.7	348.3
2	410.4	390.0	424.1
3	449.9	471.4	404.8
Delta	133.1	155.7	75.9
Row rank	2	1	3
Best choice	Level 3	Level 3	Level 2

## Data Availability

The data used to support the findings of this study are available from the corresponding authors upon request.

## References

[1] Rosenthal S., Maa F., Kamaliev M., Hahn M., Tekkaya A.E. (2020). Lightweight in Automotive Components by Forming Technology. Automot. Innov..

[2] Li H., Tian J., Fei W., Han Z., Tao G., Xu Y., Xu X., Tao J. (2019). Spring-back and failure characteristics of roll bending of GLARE laminates. Mater. Res. Express.

[3] Li H., Wang H., Alderliesten R., Xiang J., Lin Y., Xu Y., Zhao H., Tao J. (2020). The residual stress characteristics and mechanical behavior of shot peened fiber metal laminates based on the aluminium-lithium alloy. Compos. Struct..

[4] Chen Y.Z., Zhao Y., Wang H. (2020). Research progress on lightweight design and technology of fiber reinforced plastics components in automobile industry. Cailiao Gongcheng J. Mater. Eng..

[5] Sugiman S., Crocombe A.D., Katnam K.B. (2011). Investigating the static response of hybrid fibre-metal laminate doublers loaded in tension. Compos. Part B-Eng..

[6] Devi G.R., Palanikumar K. (2019). Analysis on drilling of woven glass fibre reinforced aluminium sandwich laminates. J. Mater. Res. Technol..

[7] Lin Y., Liu C., Li H., Jin K., Tao J. (2018). Interlaminar failure behavior of GLARE laminates under double beam five-point-bending load. Compos. Struct..

[8] Li H., Lu Y., Xiang J., Han Z., Xu X., Wang H., Li H., Tao J. (2020). Residual stresses and failure behavior of GFRP/Al-Li laminates after single and multiple shot’s indentation under quasi-static. Compos. Part A-Appl. Sci. Manuf..

[9] Li H., Hu Y., Fu X., Zheng X., Liu H., Tao J. (2016). Effect of adhesive quantity on failure behavior and mechanical properties of fiber metal laminates based on the aluminum-lithium alloy. Compos. Struct..

[10] Bellini C., Di Cocco V., Iacoviello F., Sorrentino L. (2019). Performance evaluation of CFRP/Al fibre metal laminates with different structural characteristics. Compos. Struct..

[11] Ostapiuk M., Bienias J., Surowska B. (2018). Analysis of the bending and failure of fiber metal laminates based on glass and carbon fibers. Sci. Eng. Compos. Mater..

[12] Carrillo J.G., Cantwell W.J. (2009). Mechanical properties of a novel fiber-metal laminate based on a polypropylene composite. Mech. Mater..

[13] Fu X., Tang X., Hu Y., Li H., Tao J. (2016). Effect of different lay-ups on the microstructure, mechanical properties and neutron transmission of neutron shielding fibre metal laminates. J. Nucl. Mater..

[14] Bhat R., Mohan N., Sharma S., Pratap A., Keni A.P., Sodani D. (2019). Mechanical testing and microstructure characterization of glass fiber reinforced isophthalic polyester composites. J. Mater. Res. Technol..

[15] Rajabi A., Kadkhodayan M., Ghanei S. (2018). An investigation into the flexural and drawing behaviors of GFRP-based fiber-metal laminate. Mech. Adv. Mater. Struct..

[16] Li H., Xu Y., Hua X., Liu C., Tao J. (2018). Bending failure mechanism and flexural properties of GLARE laminates with different stacking sequences. Compos. Struct..

[17] Ud Din I., Hao P., Franz G., Panier S. (2018). Elastoplastic CDM model based on Puck’s theory for the prediction of mechanical behavior of Fiber Reinforced Polymer (FRP) composites. Compos. Struct..

[18] Din I.U., Panier S., Hao P., Franz G., Bijwe J., Hui L. (2019). Finite element modeling of indentation and adhesive wear in sliding of carbon fiber reinforced thermoplastic polymer against metallic counterpart. Tribol. Int..

[19] Yeom K.M., Lee J. (2015). Finite Element Analysis of Large Deformation of Fiber Metal Laminates Under Bending for Stress-Strain Prediction. Trans. KSME A.

[20] Dariushi S., Sadighi M. (2013). A Study on Flexural Properties of Sandwich Structures with Fiber/Metal Laminate Face Sheets. Appl. Compos. Mater..

[21] Jin K., Wang H., Tao J., Du D. (2019). Mechanical analysis and progressive failure prediction for fibre metal laminates using a 3D constitutive model. Compos. Part A-Appl. Sci. Manuf..

[22] Zhou W., Wang Q., Ling W., He L., Tang Y., Wu F., Liao J., Hui K.S., Hui K.N. (2014). Characterization of three- and four-point bending properties of porous metal fiber sintered sheet. Mater. Des..

